# Testing multiple hypotheses on the colour change of treefrogs in response to various external conditions

**DOI:** 10.1038/s41598-023-31262-y

**Published:** 2023-03-14

**Authors:** Chohee Park, Seongsoo No, Sohee Yoo, Dogeun Oh, Yerin Hwang, Yongsu Kim, Changku Kang

**Affiliations:** 1 Department of Biosciences, Mokpo National University, Cheonggye, Muan, Jeollanamdo 58554 South Korea; 2 Department of Agricultural Biotechnology, Seoul National University, Seoul, 08826 South Korea; 3 Research Institute of Agricultural and Life Sciences, Seoul National University, Seoul, 08826 South Korea

**Keywords:** Herpetology, Animal behaviour, Animal physiology, Evolutionary ecology

## Abstract

Amphibians are famous for their ability to change colours. And a considerable number of studies have investigated the internal and external factors that affect the expression of this phenotypic plasticity. Evidence to date suggests that thermoregulation and camouflage are the main pressures that influence frogs’ adaptive colour change responses. However, certain gaps in our knowledge of this phenomenon remain, namely: (i) how do frogs adjust their colour in response to continuously changing external conditions?; (ii) what is the direction of change when two different functions of colour (camouflage and thermoregulation) are in conflict?; (iii) does reflectance in the near-infrared region show thermally adaptive change?; and (iv) is the colour change ability of each frog an individual trait (i.e., consistent within an individual over time)? Using *Dryophytes japonicus* (Hylidae, Hyla), we performed a series of experiments to answer the above questions. We first showed that frogs’ responses to continuously-changing external conditions (i.e., background colour and temperature) were not linear and limited to the range they experience under natural conditions. Second, when a functional conflict existed, camouflage constrained the adaptive response for thermoregulation and vice versa. Third, though both temperature and background colour induced a change in near-infrared reflectance, this change was largely explained by the high correlation between colour (reflectance in the visible spectrum) and near-infrared reflectance. Fourth, within-individual variation in colour change capacity (i.e., the degree of colour change an individual can display) was lower than inter-individual variation, suggesting individuality of colour change capacity; however, we also found that colour change capacity could change gradually with time within individuals. Our results collectively reveal several new aspects of how evolution shapes the colour change process and highlight how variation in external conditions restricts the extent of colour change in treefrogs.

## Introduction

Colour changes are widespread in various animal taxa^1–3^. This phenotypic plasticity can confer selective advantages to animals in heterogeneous environmental conditions since it allows animals to dynamically change to a form that performs better under different circumstances^4,5^. For instance, camouflaged animals can experience survival benefits under visually heterogeneous habitats by changing their skin colour to match their current background substrate^4,6,7^. Likewise, ectothermic animals that temporally experience various thermal conditions can modulate the amount of light they absorb by changing their skin colour to maintain an optimal body temperature^8–10^. The colour change process can be rapid (in seconds, minutes, or hours) through the aggregation or dispersion of pigment granules within chromatophores, while it can also take longer (over days to months) through changes in the number of pigment cells^1,6^.

Amphibians have long been celebrated for their colour changing ability^11,12^. Their colour change is primarily mediated by physiological processes involving the distribution of pigmented organelles^13^. A considerable amount of previous work has highlighted the internal and external factors that affect anuran colour change processes^14^. Many frogs change their dorsal colour in response to a change in background colours: darker backgrounds induce frogs to darken their colour and lighter backgrounds induce frogs to become lighter^15,16^. In consequence, background-induced colour change reinforces camouflage through better background matching^17^. Ambient temperature is another key external element that induces anuran colour change^15^. Because of their ectothermic nature, thermoregulation is critical for their activities. Because dark colours absorb more light energy than light colours, becoming darker is advantageous in colder environments and vice versa. The accumulated evidence across a diverse groups of frogs corroborates this prediction, confirming the thermoregulatory role of anuran colour change^15,18,19^. Additionally, other factors can affect these processes, including the degree of illumination, dehydration, and intra-sexual signalling^18,20,21^.

Nonetheless, there are certain gaps in our knowledge of anuran colour change processes. First, most previous studies have tested each individual only once (which is desirable to avoid pseudo-replication), but have not tested the same individual repeatedly under the same condition. Consequently, it is uncertain whether individual variation in colour change ability reflects individuality or circumstantial differences, especially in species with high intra-specific variation^17,22^. Second, frogs’ responses to external factors have mostly only been tested under a few discrete sets of conditions (e.g., black/white backgrounds or 15/25 °C temperature treatments)^15,22^. However, many frogs experience a continuum of environmental conditions, and their response to changes in more natural external conditions has not yet been demonstrated^23^. Third, frogs’ responses under circumstances in which two different functions of colouration are in conflict (e.g., camouflage and thermoregulation) are not well understood^24^. For instance, frogs resting on brightly-coloured substrates on a cold day should adopt lighter colouration for camouflage, but darker colouration to improve thermoregulation. Lastly, frogs’ colour change ability has been exclusively tested in animal-visible wavebands (300–700 nm), but hardly under other spectrums of light, such as the near-infrared range (NIR; 700–2500 nm). The change in NIR reflectance has implications for thermoregulation because more than 50% of solar radiation lies within the NIR region; thus, a change in NIR reflectance can have a significant impact on the amount of light energy that is absorbed^25^. Only recently, studies have begun to consider the adaptive significance of animal reflectance in the NIR region^8,26^. However, evidence of NIR reflectance change remains scarce and is limited to a few groups of animals such as lizards^8^.

Here, we studied the Japanese tree frog, *Dryophytes japonicus* (Hylidae, Anura; formerly considered *Hyla japonica*^27^), to tackle multiple questions on their colour change processes. *D. japonicus* changes its dorsal colour rapidly (in two hours) in response to both background colour and temperature^17,28^. *D. japonicus* also shows high intra-specific variation in colour change ability^17^. We conducted a series of experiments to examine the following questions with respect to their colour change processes: (i) how do Japanese tree frogs change their dorsal colour in response to fine-scale changes in external conditions? (ii) how do they change colour when adaptive conditions for camouflage conflict with those for thermoregulation? (iii) does their colour change accompany thermally-adaptive changes in NIR reflectance? (iv) is the colour change capacity of individual *D. japonicus* consistent over time? (i.e., is colour change capacity an individual trait?).

## Methods

### General explanations of colour change trials

All experimental procedures were approved by the Mokpo National University Institutional Animal Care & Use Committee (approval number: MNU-IACUC-2019–008). All methods were performed in accordance with the guidelines for the use of animals in research and teaching^29^. This study is reported in accordance with ARRIVE guidelines^30^. We used 32 locally-purchased *D. japonicus* that were originally collected near Yeonggwang, South Korea (N35.26°, E126.52°) for the experiment in 2019. These frogs were used for all experiments except for the individuality experiment. We purchased an additional 20 individuals for the individuality experiment performed in 2021. Each frog was kept independently in a plastic cage at 25 °C with a photoperiod of 12L:12D. We provided water and food (calcium and vitamin D-powdered mealworms and juvenile crickets) ad libitum. All colour change trials were conducted in a temperature/humidity chamber (HB-303DH, Hanbaek, South Korea) between 0900 and 1800. We maintained 70% humidity in the chamber during the trials (the temperature depended on the experiment type; see below). Fluorescent lamps were attached along the inner side of the chamber. Before conducting all experiments, we confirmed that the intensity of the light illuminated into each experimental container (measured in lux; see below) was similar (all within ± 5% differences) using a photometer.

During the colour change trials, each frog was kept in a cylindrical container (experimental arena; 14.5 cm radius × 7 cm height) and remained in the chamber for 2 h. Two hours has been shown to be sufficient for *D. japonicus* to complete a colour change process^17^. We covered the inside of the arena using one of the grey-toned papers we created (see below), and a lid covered the top of the arena. The lid was transparent, with wire mesh in the centre area that allowed airflow. After spending 2 h in the chamber, we immediately moved each frog to the photographic zone and photographed them. We placed a cylindrical container (same shape as the container used for the colour change trial, with the inside covered by grey-toned papers) in the centre of the photographic zone, in which we placed each frog to photograph its dorsum. Two bulbs illuminated the photographic zone (True-Light 23 W 5500 K, True-light International, Frankfurter), which were placed approximately 80 cm above the container. For near-infrared photography, we placed an additional bulb above the container (Halogen work light 400 W, Korea Electric, Bucheon). All photography was performed using a full-spectrum converted Nikon D7000 camera (Lifepixel, Mukilteo, WA; transmission range up to 1050 nm) equipped with a Jenoptik UV–VIS-IR 60 mm Apo Macro Lens (transmission waveband: 290–1500 nm). We attached either a Baader UV/IR-CUT Filter that permits human visible range (400 – 680 nm) or a Baader IR-Pass Filter that permits near-infrared range (670–1050 nm) for photography. We did not photograph frogs in the ultraviolet range because ultraviolet reflection is negligible in *D. japonicus*^17^. A 99% reflectance standard (WS-1-SL, Ocean Optics, Dunedin, FL) was placed next to the container and photographed together for image calibration. We saved all images in raw format. After completing all experiments, we released the frogs in the location where they were initially collected.

### Colour change in response to fine-scale changes in external conditions

In this experiment, we used various background lightness and temperature conditions to examine the frogs’ response to external conditions. For background lightness manipulation, we used eight different grey colours. To create these colours, we first printed white (non-printed) and black colours (R = G = B = 0) using Xerox Premium Nevertear Paper and photographed both papers alongside a 99% reflectance standard in raw format. Then, we converted the images to linearised reflectance TIFF images by removing the non-linearity of the images (using DcRaw v. 9.27^31^) and rescaling each colour channel value so that the 99% reflectance standard had the corresponding R, G, and B values^32^. After these processes, the R, G, and B values (0–255) in each image correspond to the reflectance (0–100%). Then, we measured the average reflectance (the average of the R, G, and B values) of the white (non-printed paper; 80% mean reflectance) and black-printed papers (4% mean reflectance) using ImageJ 1.53e (opensource program, National Institute of Health). Afterwards, we generated grey colours (equal R, G, and B values) with mean reflectance from 10 to 80% for every 10% after printing. Therefore, we created nine different grey-toned backgrounds with average reflectance values of 4, 10, 20, 30, 40, 50, 60, 70, and 80% (± 1%). We used each printed paper to cover the inside of the experimental arena and performed the colour change trials. Each frog experienced all nine background colours in a random order in three consecutive days (three treatments per day). There was at least one hour resting period between the treatment during which the frogs were placed in a plastic cage where they originally stayed with access to food and water. We maintained a 25 °C temperature during the colour change trials.

For temperature manipulation, we used the same procedure under various temperature conditions: 5, 10, 15, 20, and 25 °C. Each frog experienced all five temperature conditions in a random order in two consecutive days (three treatments on the first day and the rest two treatments on the second day). We did not test below 5 nor above 25 °C for ethical reasons; exposing frogs to these extreme conditions for 2 h without any food or water could cause harm. The tested temperature conditions were within the range that *D. japonicus* experiences in their natural habitats during the active seasons. We used non-printed papers (white) for the background colour because preliminary tests showed that colour change in response to temperature was more prominent under a white than black background.

### The interactive role of background lightness and temperature

In this experiment, we performed a 2 × 2 factorial experiment using background colour and temperature to examine (i) frogs’ responses when the demand for camouflage and thermoregulation conflict and (ii) the change in near-infrared reflectance. We used white and black colours for the background treatments and 5 and 25 °C for the temperature conditions. Each frog experienced all four colour-temperature combinations in a random order in two consecutive days (two treatments per day). When photographing each frog’s dorsum, we also photographed the frog under the near-infrared range (see above for details concerning the near-infrared photography setup).

### Individual consistency experiment

To test whether the colour change capacity of individual frogs persists over time (i.e., whether inter-individual differences in colour change capacity reflect individuality), we purchased an additional 20 frogs in February 2021; we used new frogs because those used for the above experiments had been released in 2020. We housed the frogs under 25 °C and 12L:12D conditions.

In this experiment, we primarily focused on their capacity to change colour in response to background lightness because frogs showed higher plasticity in response to changes in background lightness compared to temperature. The colour change capacity was defined as the difference in dorsal lightness when against black and white backgrounds. We tested each frog’s colour change capacity seven times on different days: the day we started the experiment (day 0), one day later, and 1, 2, 4, 9, and 17 weeks later. On each experimental day, we exposed each frog to two different background conditions (black and white) in a random order and photographed them after two hours. We maintained a 25 °C temperature during the trials. Colour capacity was calculated as the dorsal lightness of the frogs against the white background subtracted by the dorsal lightness against the black background (see "Image analysis" section for details about the measurement of dorsal lightness).

### Image analysis

We first converted all raw images into linearised TIFF images using Dcraw 9.27. Then, we generated 8-bit linear reflectance images using the 99% reflectance standard as a reference by scaling the R, G, and B values of each colour channel. After this process, each pixel’s R, G, and B values represented the reflectance ranges from 0 to 255, corresponding to a reflectance of 0%–100%. Using the linear reflectance images, we selected three random dorsal areas (but only outside the dark-patterned area) on the frog’s dorsum and measured the median R, G, and B values. Then, we calculated the median dorsal reflectance (hereafter referred to as the dorsal lightness) as the average of the measured R, G, and B values divided by 2.55. We also calculated the hue and chroma according to the methodology of Smith et al.^24^. However, hue changes were negligible, and lightness change largely predicted chroma change^17^; thus, we focused our analysis on lightness. Our primary interest was to compare the extent of dorsal colour change in frogs, not how predators or conspecifics perceive frogs’ dorsal colour; therefore, we did not assume any specific receiver visual systems for most experiments^33^. Nonetheless, we tried modelling bird vision for the background colour change experiment to test whether the change in frog dorsal colour is distinguishable from the viewpoint of birds (see Supplementary methods and results).

To measure dorsal reflectance in the near-infrared region, we first converted the raw near-infrared images into 8-bit linear reflectance TIFF images using the procedure described above. Then, we used only R channel pixel values for the analysis (after dividing them by 2.55), which showed the most sensitive responses in the near-infrared range.

### Data analysis

To compare dorsal lightness across various grey-toned backgrounds with different reflectances, we used linear mixed models (LMMs) with dorsal lightness as the response variable and background reflectance as a discrete predictor. Frog ID was used as a random factor in all mixed model analyses. Similarly, to compare dorsal lightness across the various temperature conditions, we used LMMs with dorsal lightness as the response variable and temperature condition as a discrete predictor. For temperature data, there was a noticeable linear and gradual change in frog dorsal reflectance as temperature increased; therefore, we additionally fitted another LMM with dorsal lightness as the response variable and temperature condition as a continuous predictor. For post-hoc comparisons of the LMMs, we used the Tukey’s multiple pairwise comparisons implemented in the “glht” function of the “multcomp” package^34^. We controlled for false discovery rates whenever multiple comparisons were conducted^35^.

We analysed visible-range colour and near-infrared reflectance separately for the interactive role of colour and temperature experiment. For the visible-range analysis, we fitted LMMs with visible-range dorsal lightness as the response variable, and the two treatments (background colour and temperature) as well as their interaction as predictors. For the near-infrared range analysis, we fitted additional LMMs with the same predictors, but with near-infrared range lightness as the response variable. In addition, because near-infrared lightness correlated highly with visible-range lightness^26,36^, we also analysed whether any “special” adaptations existed in near-infrared range lightness after accounting for their high correlation with visible-range lightness. To do this, we fitted another LMM using near-infrared lightness as the response variable, colour and temperature treatment and their interaction as predictors, and visible-range lightness as a covariate. When there were multiple predictors, we used the information theoretic approach for model inference^37^. We first generated all candidate models and ranked each based on the Akaike Information Criterion corrected for small sample sizes (AICc). Then, we selected models with ΔAICc < 4 and conditionally averaged (i.e., averaged over the models where the parameter appeared) the parameters of all selected models and estimated *P* values based on the Wald z statistic.

Using the same data, we also tested whether frogs with higher colour capacity in response to background lightness also showed higher colour capacity in response to temperature. We calculated each frog’s colour change capacity in response to both background (the lightness difference between when they were against black and white backgrounds) and temperature (the difference between when they were under 5 and 25 °C conditions). Then, we performed a Pearson’s correlation test on the two variables.

To analyse individual consistency in colour change capacity, we estimated intraclass correlation coefficients (ICCs)^38^. ICCs are a type of correlation, but operate on data structured as groups. Here, we tested whether each frog’s colour change capacity was similar across different trials (ICC for absolute agreement). *P* values and confidence intervals were estimated using a *F* test. Additionally, we fitted LMMs using each frog’s colour change capacity as a response, trial as a discrete predictor, and frog ID as a random factor; we performed this additional analysis to (1) extract the amount of variance explained by the random factor (individual frog ID) and (2) test whether there was a directional trend in frogs’ colour change capacity across trials. We present our results with a gradual notion of evidence^39^.

## Results

### Colour change against continuously-changing background brightness

**Figure 1:**
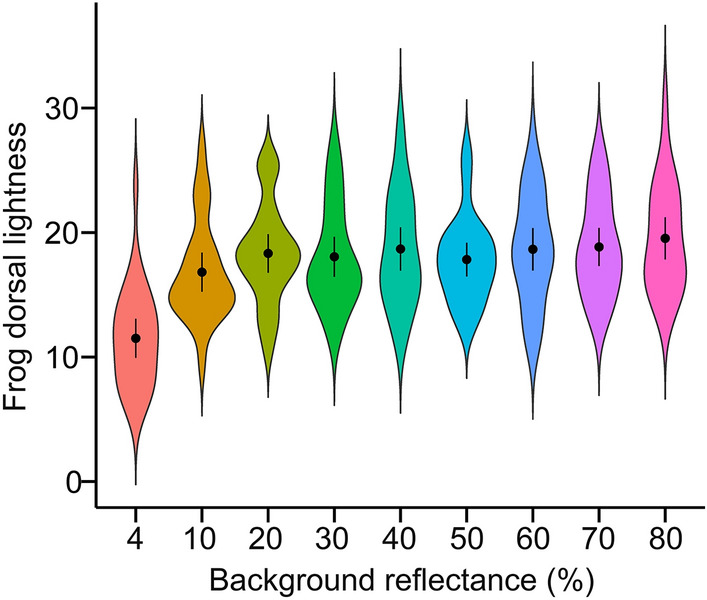
A violin plot showing the lightness of the dorsal area of *D. japonicus* against various grey-toned backgrounds. The dorsal lightness value corresponds to the mean reflectance in percentage (see "Methods" section). Points indicate means, and bars show 95% confidence intervals.

There was very strong evidence that background reflectance predicts frog dorsal lightness (Fig. 1; $\chi_{8}^{2}$ = 156.45, *P* < 0.001). Post-hoc comparisons showed that dorsal lightness against the 4% reflectance background was lower than dorsal lightness against all other backgrounds (all *P*_*adj*_ < 0.001; see Table S1 for full statistics). There was a weak to moderate evidence that dorsal lightness against the 10% reflectance background is different from that against the 40 (*P*_*adj*_ = 0.05), 60 (*P*_*adj*_ = 0.05), 70 (*P*_*adj*_ = 0.03), and 80% reflectance (*P*_*adj*_ = 0.001) backgrounds. We found no evidence of the difference between all other groups.

### Colour change against continuously-changing temperature conditions

**Figure 2:**
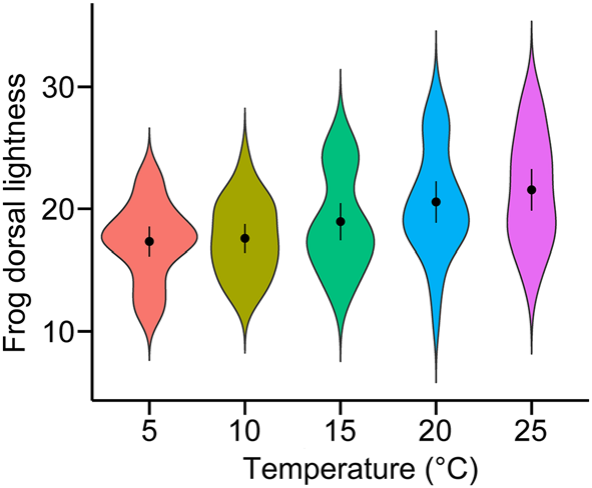
A violin plot showing the lightness of the dorsal area of *D. japonicus* under various temperature conditions against a white background. The dorsal lightness value corresponds to the mean reflectance in percentage (see “Methods” section). Points indicate means, and bars show 95% confidence intervals.

There was strong evidence for the dorsal lightness differences across the temperature treatments (Fig. 2; $\chi_{4}^{2}$ = 26.00, *P* < 0.001; see Table S2 for full statistics). Post-hoc comparisons revealed that dorsal lightness under the 5 °C condition was lower than that under both the 20 °C (*P*_*adj*_ = 0.005) and 25 °C (*P*_*adj*_ < 0.001) conditions. Similar differences were seen under the 10 °C conditions: dorsal reflectance under the 10 °C condition was lower than that under both the 20 °C (*P*_*adj*_ = 0.009) and 25 °C (*P*_*adj*_ < 0.001) conditions. Additionally, dorsal reflectance under the 15 °C condition was lower than that under the 25 °C condition (*P*_*adj*_ < 0.02). We found no evidence for the difference among all other comparisons (*P*_*adj*_ > 0.16; Table S2). When we used temperature as a continuous predictor and fit a linear model, there was strong evidence of increasing trend of dorsal lightness as the temperature increased (estimate = 0.23, s.e. = 0.05, *P* < 0.001).

### The interactive role of background lightness and temperature

**Figure 3:**
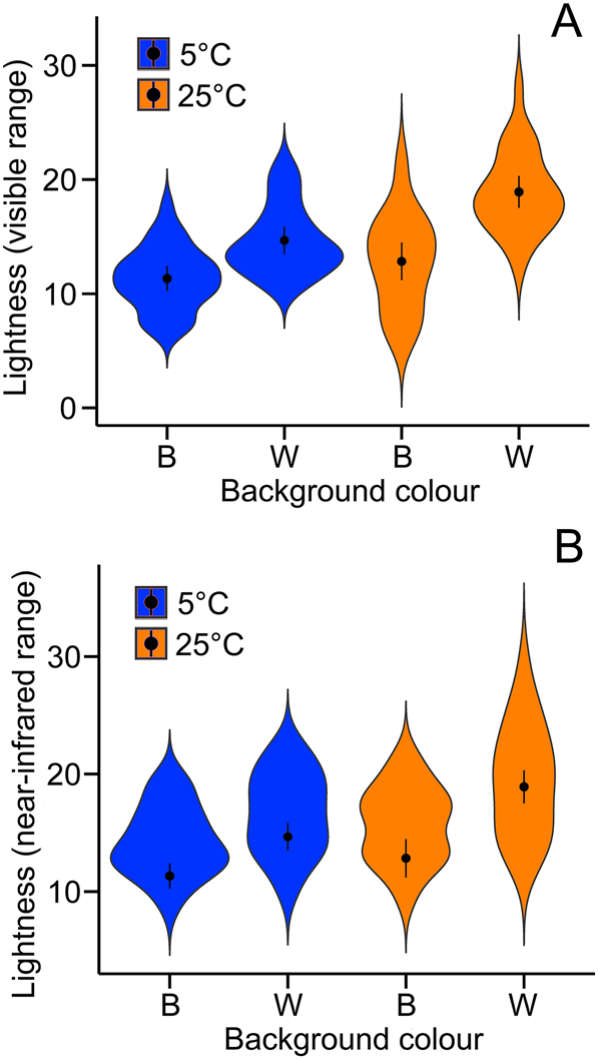
Violin plots showing the effect of two temperature and background colour (black or white) treatments on the dorsal lightness of *D. japonicus* under the visible (**A**) and near-infrared (**B**) spectrums. Points indicate means, and bars show 95% confidence intervals.

In the visible reflectance range, the data revealed strong evidence of the presence of the interaction effect between background colour and temperature on the dorsal lightness of frogs (Fig. 3a; estimate = 0.68, *t*_*81*_ = 2.71, *P* = 0.008). The interaction effect suggests that (i) the frogs’ response to background colour was stronger under the 25 °C condition than the 5 °C condition (mean difference 6.07 vs. 3.33), and (ii) the frogs’ response to temperature was stronger against white backgrounds than black backgrounds (mean difference 4.23 vs. 1.51). There were also a strong evidence of the main effect of both background colour (lighter against white backgrounds; estimate = 2.35, *t*_*81*_ = 9.29, *P* < 0.001) and temperature (lighter under the 25 °C condition; estimate = 1.43, *t*_*81*_ = 5.68, *P* < 0.001).

Colour change observed in the near-infrared range did not deviate from that in in the visible range (Fig. 3b), except that the effect of the interaction between background colour and temperature diminished. In the near-infrared range, background colour (estimate = 3.18, *t*_*81*_ = 5.19, *P* < 0.001) and temperature (estimate = 4.63, *t*_*81*_ = 7.55, *P* < 0.001) affected frog dorsal lightness, but we found no evidence that the interaction effect between background colour and temperature is present (estimate = 1.00, *t*_*81*_ = 1.63, *P* = 0.11). However, these effects appear to be principally driven by the correlation between the visible and near-infrared range lightness (average Pearson correlation coefficient *r* between visible and near-infrared range lightness across treatments = 0.42). In the model in which lightness in the visible range was included as a covariate, visible range lightness was the only variable that predicts the near-infrared range lightness (estimate =  − 0.25, *t*_*81*_ = 10.92, *P* < 0.001), and all other variables did not predict the near-infrared range lightness (all *P* > 0.22).

**Figure 4:**
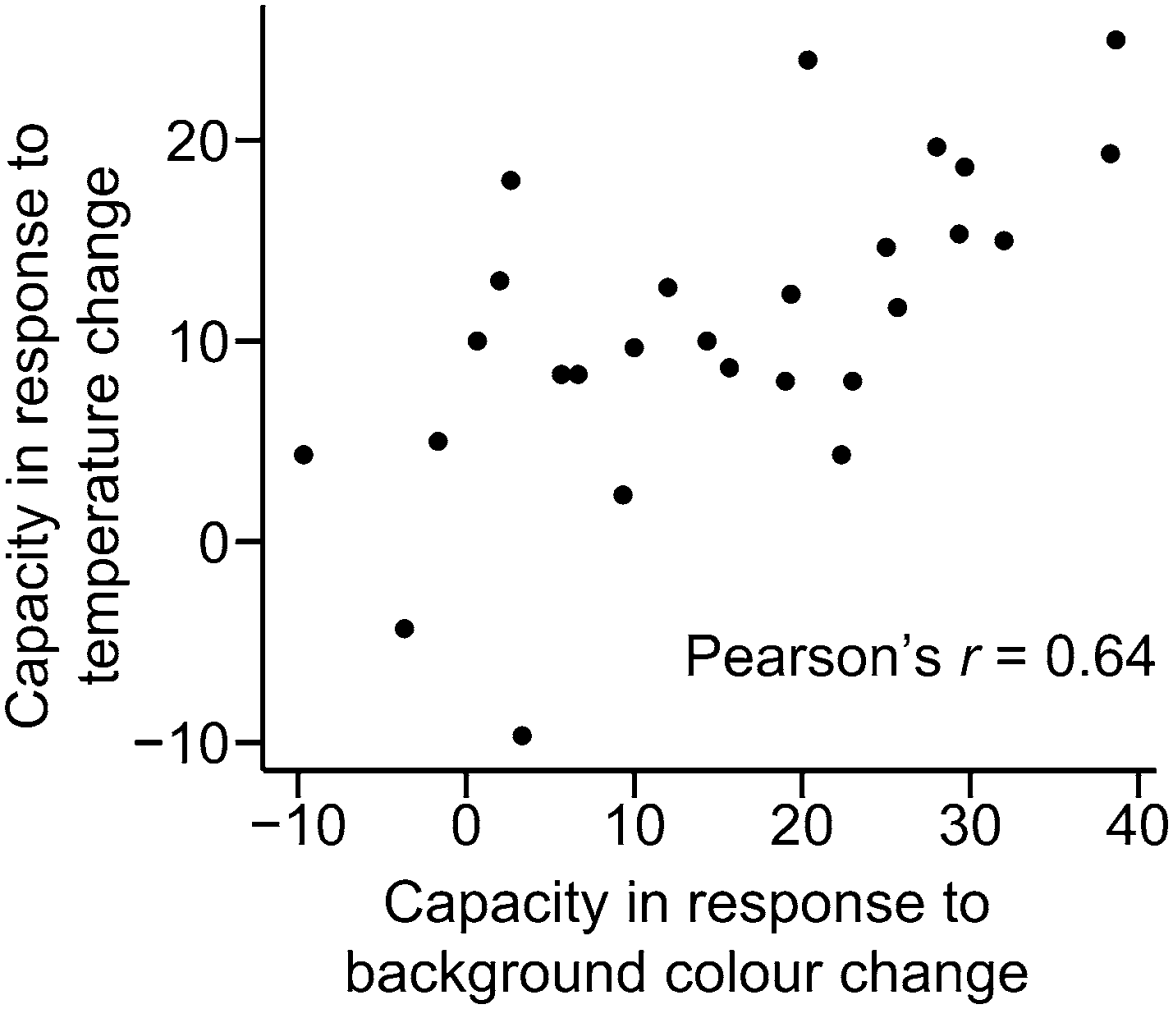
Correlation between colour change capacity in response to the change in background colour (x-axis) and temperature (y-axis).

We found very strong evidence that frogs with a higher colour change capacity against different background colours also showed a higher capacity in response to the change in temperature (Fig. 4; Pearson’s *r* = 0.64, *t*_*25*_ = 4.18, *P* < 0.001).

### Individuality

**Figure 5:**
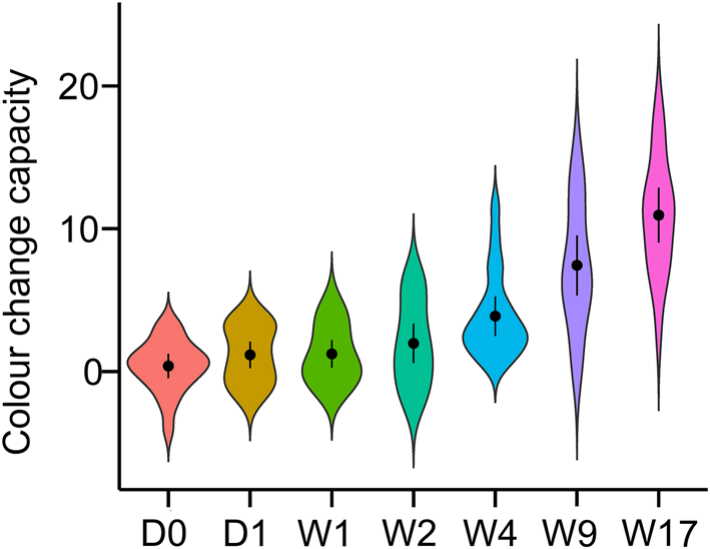
A violin plot showing the distribution of colour change capacity of the same individuals across different days. Points indicate means, and bars show 95% confidence intervals. D0 is the day that the experiment started. D: day, W: week.

Frog ID explained 41.19% of the total variation in the individuality data. Intraclass correlation tests revealed very strong evidence that the ICC deviated from 0 (estimated *ICC* = 0.64, *P* = 0.001), illustrating that within-individual variation in colour change capacity was smaller than among-individual variation. However, we also found very strong evidence that the frogs’ colour change capacity differed among the measured dates (Fig. 5; $\chi_{6}^{2}$ = 333.47, *P* < 0.001): while the results revealed no evidence for the difference in colour change capacity from D0 (day 0) to W2 (week 2), colour change capacity started to change from W4 with an increasing trend (Table S3 for post-hoc comparison statistics). Further correlation analysis of the frogs’ colour change capacity between the first and last trials demonstrated strong evidence that those who had a higher colour capacity at the beginning of the experiment (day 0) also showed a higher colour capacity at the last trial (week 17; *t*_*18*_ = 2.75, Pearson’s *r* = 0.54, *P* = 0.01).

## Discussion

In line with previous studies of amphibian colour change, *D. japonicus* changed its dorsal colour in response to both background lightness and temperature^15,17,18^; however, the degree to which they changed colours was substantially higher in response to background lightness than temperature. Our series of experiments revealed several new aspects of treefrog colour changes. First, the frogs’ dorsal lightness did not increase linearly with increasing background lightness. Instead, dorsal lightness showed an abrupt increase within a low background lightness zone (between 0 and 20%) and maintained a similar dorsal lightness when the background reflectance was greater than 20%. In contrast, the frogs’ dorsal colour gradually increased in response to temperature over broad temperature conditions from 10 to 25 °C. Second, *D. japonicus* changed their dorsal colour in both visible and near-infrared spectrum; however, their colour change in the near-infrared region was largely explained by the high correlation between visible and near-infrared dorsal reflectance, and we found no evidence for specific adaptations present in the near-infrared regions. Third, the degree to which frogs changed their colour in response to temperature depended on background lightness and vice versa. Fourth, the capacity for individual frogs to change their dorsal colour was more similar within individuals than across individuals. In addition, individuals who showed a higher colour change capacity in response to background lightness also showed a higher capacity in response to temperature. These findings support the individuality of colour change capacity. Interestingly and unexpectedly, we also found that colour change capacity can change gradually over time within individuals.

In response to background lightness, *D. japonicus* changed colours only when they were against backgrounds with a mean reflectance value less than 20%. In nature, the most widely used background substrate of tree frogs is either leaves, branches, or tree barks^40,41^; these substrates usually have average reflectance below 20% within the animal-visible spectrum, with only a few exceptions^42,43^. Thus, frogs’ responses to a lighter substrate may be selected against due to it being unnecessary. We also stress that the average reflectance of frogs remained between 10 and 20%, which is also within the background reflectance range under which they responded. Similarly, during their active season (roughly from May to October), the average daily temperature of their collection site varied from approximately 10 to 29 °C (based on 2019 data from the Korea Meteorological Administration). This range corresponds to the range under which we found frogs’ dorsal lightness to gradually increase (from 10 to 25 °C). These results suggest that the external conditions under which *D. japonicus* change colour is limited to the conditions they experience in their natural habitats.

Our results show that the effect of temperature and background lightness are not additive. The degree of colour change in response to different temperature conditions depended on the focal background lightness; temperature-induced dorsal colour change was three times more prominent against white than dark backgrounds. This finding suggests that survival costs of background mismatch may exceed the thermoregulatory benefits, especially when the frogs are against dark rather than lightly coloured backgrounds. Similarly, background-induced colour change was about twice as high when under 25 than 5 °C conditions; this implies that the pressure for thermoregulation can constrain adaptive colour change for background matching as well. Nonetheless, frogs appear to adjust their colour primarily for camouflage: when the temperature-induced colour change was limited (i.e. when the background colour was black), the mean difference in dorsal lightness under the two temperature conditions was only 1.51%. On the other hand, when background-induced colour change was limited (under 5 °C conditions), the mean lightness difference was about two times larger (3.33%). These results are in line with those of a previous study of lizards, which found that they predominantly adjust their colour for camouflage^24^.

We note here that the colour change for thermoregulation in treefrogs in natural conditions may depend on two components: response to ambient temperature and light intensity (solar radiation). Most previous studies demonstrate that frogs change their colour in response to a change in ambient temperature^15,18^. However, the evidence for the effect of light intensity is scarce. Our study also manipulated ambient temperature while controlling for light intensity. Thus, we cannot exclude the possibility that the observed colour change under different temperature conditions may be different from what happens in nature if light intensity and ambient temperature interactively affect the colour change process. One hypothesis is that the degree of temperature-induced colour change increases with light intensity because, under stronger radiation, the amount of absorbed/reflected light energy will be greater than under weaker radiation.

The individuality experiment showed that within-individual variation in colour change capacity was lower than among-individual variation. We also found that individuals with a higher colour capacity in response to background lightness also had a higher capacity in response to temperature. These suggest that the colour change capacity of *D. japonicus* is likely an individual trait. However, about 59% of the variation was not explained by individuality, and the results also showed that individual colour change capacity can change gradually over time. En masse, individual differences exist and persist, but colour change capacity is not fixed within individuals but could change depending on external factors. Then what factors do affect the colour change capacity of individuals? One hypothesis is that body condition affects colour change capacity. The frogs used for the individuality experiment were brought to the laboratory in February 2021, and used in the experiment several days after their arrival. Although we did not regularly measure it directly, all frogs showed a low body condition (commonly defined as a ratio between body mass and a linear measure of size) at the beginning of the experiment judged by their slim body shape (personal observations by all authors; Figure. S1). However, they gradually gained weight as the experiment progressed (Figure. S1); this increase in body condition corresponds to their increasing colour change capacity. The increased body condition may have helped frogs afford the energy costs of colour change^44^. One can also argue that the season may affect colour change capacity^45^. The individuality experiment was performed from February to June 2021. Since the active season of *D. japonicus* is from May to October, their colour change capacity may be adapted to being more strongly expressed during non-brumation seasons, potentially explaining the observed gradual increase in colour plasticity. However, we consider this possibility to be low because (i) the housing conditions were constant during the individuality experiment (24 °C, 60% humidity, 12L:12D), such that frogs would be unable to perceive seasonal changes without internal mechanisms, and (ii) the background lightness experiment (though using another batch of frogs) was also performed during the non-active season (February to March, 2020), but these frogs showed consistently higher colour change capacity. The factors that affect colour change capacity of *D. japonicus* remain to be tested.

While adaptations in near-infrared reflectance for thermoregulation have been demonstrated in several taxa^8,26^, we found no specific adaptations in *D. japonicus*. Although the colour change of *D. japonicus* also affected reflectance in the near-infrared range, the change in the near-infrared range is primarily explained by the high correlation between visible and near-infrared reflectance. It has been suggested that thermoregulation may not be the primary function of near-infrared reflection in treefrogs because the change in body surface temperature induced by the near-infrared reflectance difference is only little^46^. Likewise, the observed change in near-infrared reflectance through colour change in *D. japonicus* may simply be a by-product of colour change in the visible spectrum.

In summary, our study highlights that (i) the range of background lightness and temperature under which *D. japonicus* change colour falls within the range that they experience in their natural habitat, (ii) the demand for camouflage can restrict the adaptive response for thermoregulation and vice versa, and the degree to which *D. japonicus* change their dorsal colour was greater in response to the change in background lightness than temperature change, iii) *D. japonicus*’s colour change capacity is likely an individual trait though the capacity is not fixed but can change gradually within individuals, and (iv) the colour change of *D. japonicus* accompanies correlative changes in near-infrared reflectance. These new findings broaden our insight into amphibian colour change and raise further questions, such as the evolutionary mechanism that maintains individual variation in colour change capacity, and the inheritance and ontogenic expression of colour change capacity in amphibians.

## Supplementary Information

Supplementary Information.

## Data Availability

The datasets analysed during the current study are available in the figshare repository (https://doi.org/10.6084/m9.figshare.21739292.v1).
