# Supplementary material for: Testing multiple hypotheses on the colour change of treefrogs in response to various external conditions
Source: Sci Rep. 2023 Mar 14;13:4203. doi: 10.1038/s41598-023-31262-y (PMC10015036; doi:10.1038/s41598-023-31262-y)
Supplement: Supplementary file 1 — Supplementary Information. [file 41598_2023_31262_MOESM1_ESM.pdf]

Supplementary Information for the paper:

Testing multiple hypotheses on the colour change of treefrogs in response to various external conditions

Chohee Park, Seongsoo No, Sohee Yoo, Dogeun Oh, Yerin Hwang, Yongsu Kim, Changku Kang

Table S1. Post-hoc comparison results of colour change experiment in response to background lightness. Comparisons with P > 0.05 were highlighted in bold.

Comparison (background reflectance %)	Estimate	z	adjusted P
**4 vs 10**	**5.32**	**6.93**	**<0.001**
**4 vs 20**	**6.82**	**8.88**	**<0.001**
**4 vs 30**	**6.55**	**8.53**	**<0.001**
**4 vs 40**	**7.18**	**9.35**	**<0.001**
**4 vs 50**	**6.33**	**8.24**	**<0.001**
**4 vs 60**	**7.15**	**9.32**	**<0.001**
**4 vs 70**	**7.34**	**9.56**	**<0.001**
**4 vs 80**	**8.03**	**10.46**	**<0.001**
10 vs 20	1.5	1.96	0.129
10 vs 30	1.23	1.61	0.244
10 vs 40	1.86	2.42	0.051
10 vs 50	1.01	1.31	0.358
10 vs 60	1.83	2.39	0.051
**10 vs 70**	**2.02**	**2.63**	**0.03**
**10 vs 80**	**2.71**	**3.53**	**0.002**
20 vs 30	-0.27	-0.35	0.815
20 vs 40	0.35	0.46	0.773
20 vs 50	-0.49	-0.65	0.645
20 vs 60	0.33	0.43	0.773
20 vs 70	0.52	0.68	0.642
20 vs 80	1.21	1.58	0.244
30 vs 40	0.63	0.82	0.575
30 vs 50	-0.22	-0.29	0.84
30 vs 60	0.6	0.78	0.577
30 vs 70	0.79	1.03	0.456
30 vs 80	1.48	1.93	0.129
40 vs 50	-0.85	-1.11	0.439
40 vs 60	-0.02	-0.03	0.976
40 vs 70	0.16	0.21	0.855
40 vs 80	0.85	1.11	0.439
50 vs 60	0.83	1.08	0.441
50 vs 70	1.01	1.32	0.358
50 vs 80	1.7	2.22	0.073
60 vs 70	0.19	0.24	0.855
60 vs 80	0.88	1.14	0.439
70 vs 80	0.69	0.9	0.53

Table S2. Post-hoc comparison results of colour change experiments in response to temperature. Comparisons with P > 0.05 were highlighted in bold.

Comparison	Estimate	z	adjusted P
5 vs 10 °C	0.26	0.25	0.801
5 vs 15 °C	1.62	1.58	0.163
**5 vs 20 °C**	**3.23**	**3.16**	**0.005**
**5 vs 25 °C**	4.22	4.14	**0**
10 vs 15 °C	1.36	1.33	0.229
**10 vs 20 °C**	**2.97**	**2.91**	**0.009**
**10 vs 25 °C**	**3.96**	**3.89**	**0.001**
15 vs 20 °C	1.61	1.58	0.163
**15 vs 25 °C**	**2.61**	**2.55**	**0.021**
20 vs 25 °C	0.99	0.98	0.366

Table S3. Post-hoc comparison results of colour change experiments in response to temperature. Comparisons with P > 0.05 were highlighted in bold.

Comparison	Estimate	z	adjusted P
D0 vs D1	0.78	1.05	0.94292
D0 vs W1	0.85	1.14	0.91686
D0 vs W2	1.58	2.12	0.3383
**D0 vs W4**	**3.5**	**4.69**	**0.001**
**D0 vs W9**	**7.05**	**9.45**	**0.001**
**D0 vs W17**	**10.56**	**14.16**	**0.001**
D1 vs W1	0.07	0.09	1
D1 vs W2	0.8	1.08	0.93489
**D1 vs W4**	**2.71**	**3.64**	**0.00507**
**D1 vs W9**	**6.27**	**8.4**	**0.001**
**D1 vs W17**	**9.78**	**13.11**	**0.001**
W1 vs W2	0.74	0.99	0.95682
**W1 vs W4**	**2.65**	**3.55**	**0.0072**
**W1 vs W9**	**6.2**	**8.31**	**0.001**
**W1 vs W17**	**9.71**	**13.02**	**0.001**
W2 vs W4	1.91	2.56	0.13767
**W2 vs W9**	**5.46**	**7.32**	**0.001**
**W2 vs W17**	**8.97**	**12.03**	**0.001**
**W4 vs W9**	**3.55**	**4.76**	**0.001**
**W4 vs W17**	**7.06**	**9.47**	**0.001**
**W9 vs W17**	**3.51**	**4.71**	**0.001**

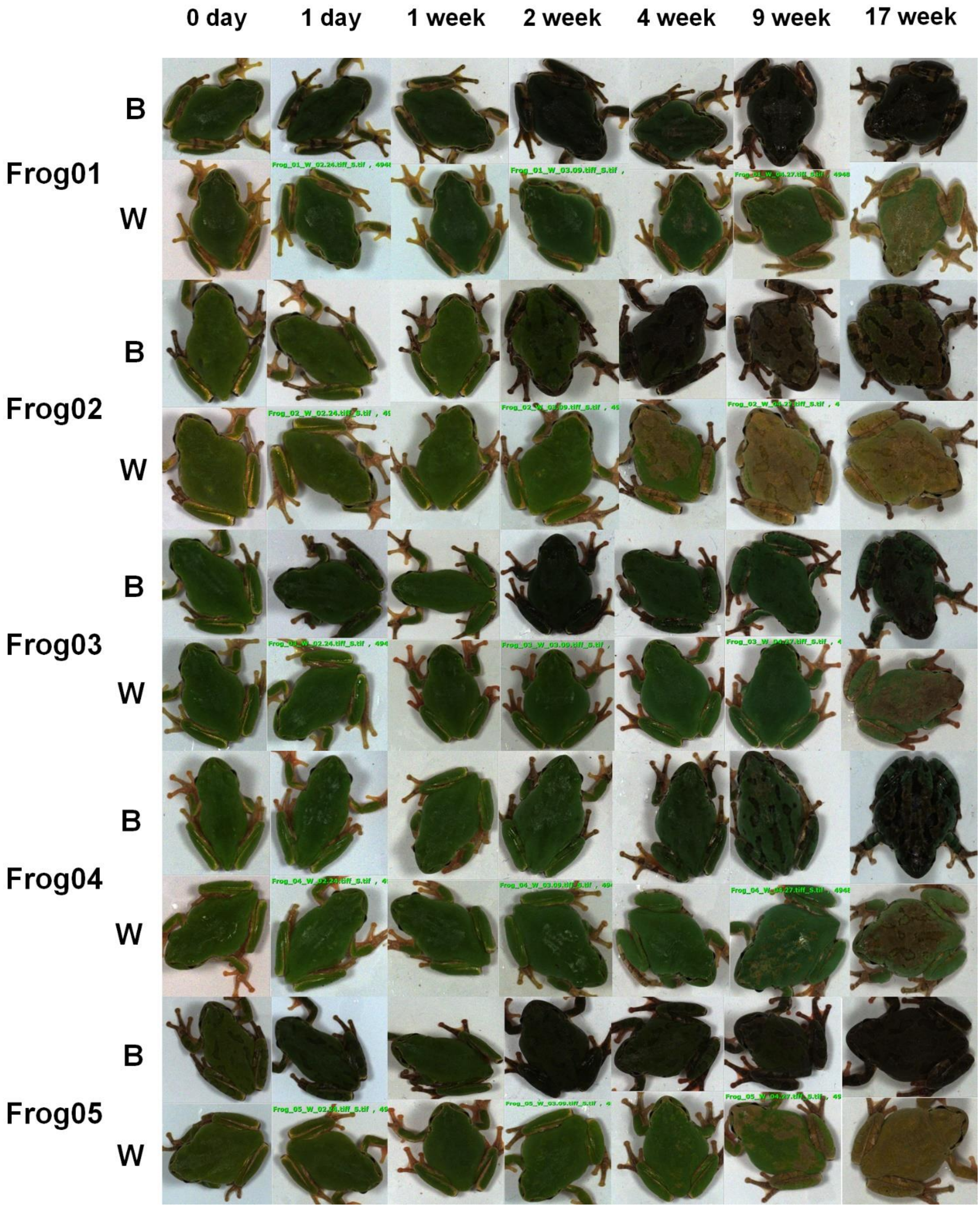

Figure S1. Example photos of frogs used in the individuality experiment. Notably, colour change capacity and their weight increased correlatively as time passed. The causality of this relationship remains to be tested. B: individuals that were placed against a black background for two hours. W: the same individuals that were placed against a white background for two hours.

Supplementary methods

Further analysis of the background colour experiment by applying predator vision modelling

Here, we examined whether the dorsal colour change of *D. japonica* yielded perceptible differences in colour from the viewpoint of predators. We first converted raw images to cone-catch images using the micaToolbox implemented in ImageJ 1.52a (open-source program, National Institute of Health, USA)[1]. We utilised a widely used receptor-noise limited model assuming blue tit (*Cyanistes caeruleus*) visual system[2]. Then we estimated the chromatic (derived from single cone catches) and achromatic (derived from double cone catches) just noticeable differences (JNDs) of frog dorsal colours among different background treatments. Due to the lack of UV images, we used trichromatic modelling using the information from only long-, medium-, and short-wavelength sensitive cones. The two colours with a JND value < 1 are considered to be indistinguishable. We assumed the Weber noise for long-wavelength photoreceptors as 0.05, and the relative proportion of the different cone types in the retina as 1.9: 2.7: 2.7 for the short-, medium-, and long-wavelength sensitive cone cells[2,3]. Because (i) our background treatments differed not in the chromatic component but only in the achromatic component, and (ii) the dorsal colour change of frogs was dominated by achromatic changes, we only analysed achromatic JNDs. Because the frog dorsal colours against higher than 30% reflectance backgrounds were not statistically different from those colours against 20 % reflectance background, we only compared the dorsal colours against 0, 10, and 20% reflectance backgrounds where the dorsal colour differences were observed.

We examined the validity of null hypothesis that the estimated achromatic JNDs are not different from one. The alternative hypothesis was that the estimated achromatic JNDs are larger than 1. We used one-sample t-test to test the hypothesis.

Results

The change in frog dorsal lightness against both 10 and 20% reflectance backgrounds were readily distinguishable from those against 0% reflectance background (JND > 1, Table S4). However, the difference between 10 and 20% reflectance backgrounds were indistinguishable (Table S4).

Table S4. The results of the colour discrimination model comparing frog dorsal colours among different background treatments. Only achromatic components (estimated from double cone catches) were analysed. JND: just noticeable difference.

Comparison	Mean JND ± SEM	*t*	*df*	*P*
0 vs 10 % reflectance background	1.71 ± 0.22	3.20	27	0.002
0 vs 20 % reflectance background	2.06 ± 0.21	5.05	27	<0.001
10 vs 20 % reflectance background	0.65 ± 0.10	-3.41	27	1

1. Troscianko, J. & Stevens, M. Image calibration and analysis toolbox – a free software suite for objectively measuring reflectance, colour and pattern. *Methods Ecol. Evol.* **6**, 1320–1331 (2015).

2. Vorobyev, M. & Osorio, D. Receptor noise as a determinant of colour thresholds. *Proc. R. Soc. London B Biol. Sci.* **265**, 351–358 (1998).

3. Hart, N. S. The visual ecology of avian photoreceptors. *Prog. Retin. Eye Res.* **20**, 675–703 (2001).
